# Fundamentals to therapeutics: Epigenetic modulation of CD8^+^ T Cell exhaustion in the tumor microenvironment

**DOI:** 10.3389/fcell.2022.1082195

**Published:** 2023-01-04

**Authors:** Maja K. Blake, Patrick O’Connell, Yasser A. Aldhamen

**Affiliations:** Department of Microbiology and Molecular Genetics, College of Osteopathic Medicine, Michigan State University, East Lansing, MI, United States

**Keywords:** CD8^+^ T cell, epigenetics, tumor microenvironment, TOX, SLAMF7, PD-1, HDAC, ATAC-seq

## Abstract

In the setting of chronic antigen exposure in the tumor microenvironment (TME), cytotoxic CD8^+^ T cells (CTLs) lose their immune surveillance capabilities and ability to clear tumor cells as a result of their differentiation into terminally exhausted CD8^+^ T cells. Immune checkpoint blockade (ICB) therapies reinvigorate exhausted CD8^+^ T cells by targeting specific inhibitory receptors, thus promoting their cytolytic activity towards tumor cells. Despite exciting results with ICB therapies, many patients with solid tumors still fail to respond to such therapies and patients who initially respond can develop resistance. Recently, through new sequencing technologies such as the assay for transposase-accessible chromatin with sequencing (ATAC-seq), epigenetics has been appreciated as a contributing factor that enforces T cell differentiation toward exhaustion in the TME. Importantly, specific epigenetic alterations and epigenetic factors have been found to control CD8^+^ T cell exhaustion phenotypes. In this review, we will explain the background of T cell differentiation and various exhaustion states and discuss how epigenetics play an important role in these processes. Then we will outline specific epigenetic changes and certain epigenetic and transcription factors that are known to contribute to CD8^+^ T cell exhaustion. We will also discuss the most recent methodologies that are used to study and discover such epigenetic modulations. Finally, we will explain how epigenetic reprogramming is a promising approach that might facilitate the development of novel exhausted T cell-targeting immunotherapies.

## Introduction

Immunotherapy is a promising new treatment for cancer, one which harnesses a patient’s own immune system to target tumor cells. These therapies aim to boost anti-tumor activity of cytotoxic immune cells, such as cytotoxic CD8^+^ T cells and Natural Killer (NK) cells. During acute infections, naïve CD8^+^ T cells undergo robust proliferation and clonal expansion to differentiate into effector and memory CD8^+^ T cells. In contrast, in cancer and during chronic infections persistent antigen stimulation abrogates the development of memory T cells and T cells become exhausted. In cancer, immunotherapies attempt to enhance CD8^+^ T cell effector function by targeting various inhibitory receptors on the cells’ surfaces. For example, anti-programmed death protein 1 (PD-1) monoclonal antibodies are exceptional for PD-1 receptor inhibition, thereby promoting anti-tumor CD8^+^ T cell activity (Hellmann et al., 2019; Hellmann and Ramalingam, 2020). Anti-PD-1 therapies such as pembrolizumab and nivolumab were first approved for melanoma (Wolchok et al., 2013) and have since shown exceptional results in other cancers as well (Tawbi et al., 2018; Doki et al., 2022). Similarly, chimeric antigen receptor-T (CAR-T) cell therapy harnesses T cell-mediated anti-tumoral immune activity, an immune therapy approach that optimizes T cell activity against tumor cells. As promising as this therapy is for specific types of hematological malignancies, CAR-T therapy are less effective against solid tumors, and are also associated with some significant safety concerns (Gust et al., 2020; Rubin and Vaitkevicius, 2021). Specifically, in both CAR-T and immune checkpoint blockade (ICB) such as anti-PD-1 therapy, resistance to treatment remains a significant issue that limits their efficacy and utilization for a large subset of cancer patients (Gust et al., 2020; Rubin and Vaitkevicius, 2021). Therefore, defining the mechanisms that mediate resistance to CTL-based immunotherapies is of upmost importance (Sade-Feldman et al., 2018; Klemen et al., 2019; Lu et al., 2019).

One mechanism of resistance to T cell-targeting immunotherapies is loss of T cell effector activity (Pauken et al., 2016; Balança et al., 2021), whereby dysfunctional/exhausted CD8^+^ T cells fail to adequately clear cancer cells. Recently, epigenetic alterations were found to reinforce the differentiation of naive T cells to the exhausted state (Yang and Wang, 2021; Belk et al., 2022; Chen et al., 2022). Therefore, we and others believe that CD8^+^ T cells can be re-sensitized to ICB therapy by modulating the activity of specific epigenetic factors that mediate CD8^+^ T cell exhaustion. Here, we will describe effector and exhausted CD8^+^ T cell differentiation in the tumor microenvironment, explore transcription factors and genomic alterations reinvigorating such differentiation, and examine therapies which target these epigenetic pathways as a means to enhance the efficacy of ICB therapy.

## T cell development in the TME

**FIGURE 1:**
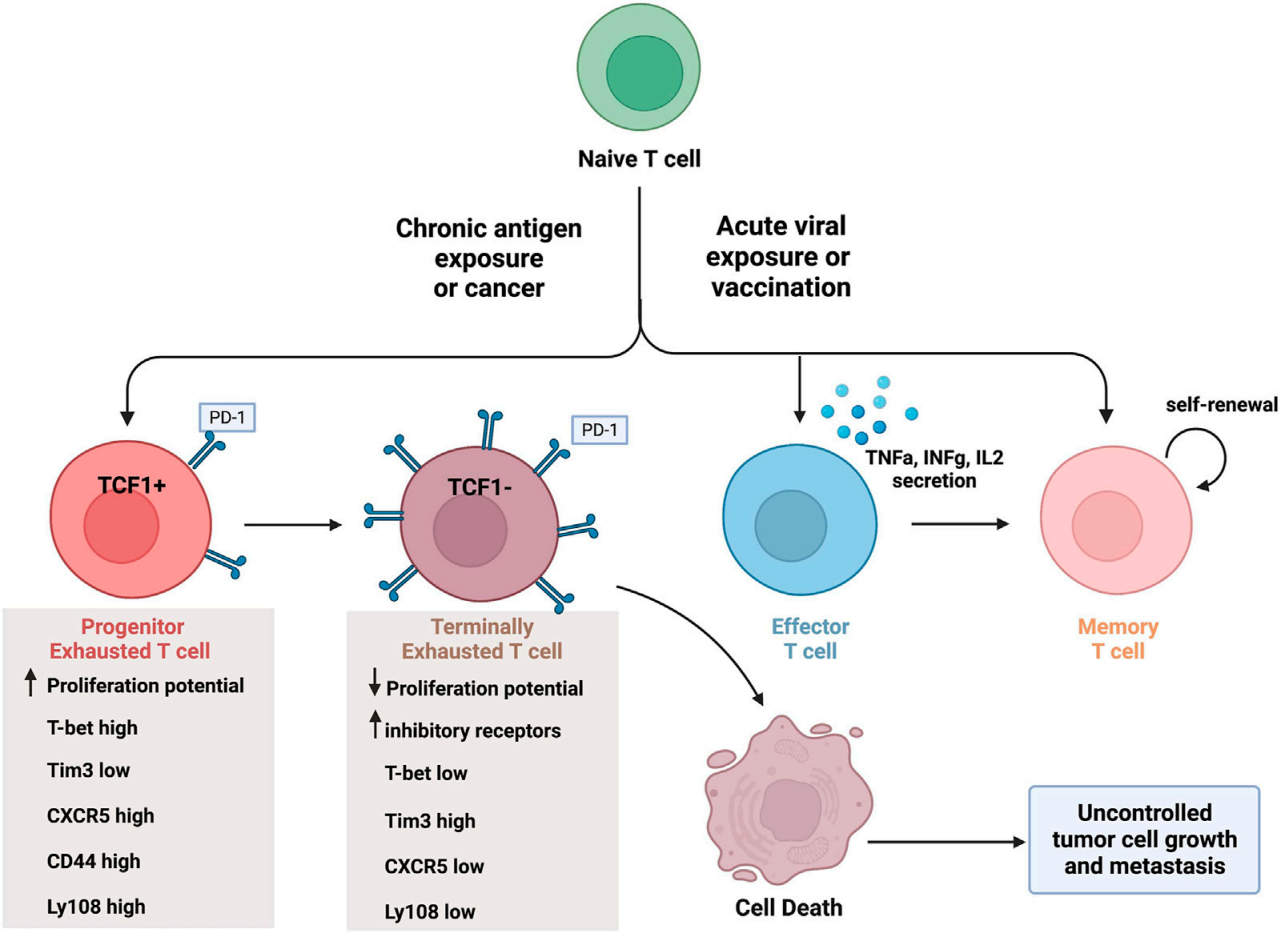
CD8^+^ T cell differentiation in the tumor microenvironment and during acute infection. Naïve T cells can differentiate into various CD8^+^ T cell subsets upon acute antigen exposure, and this varies in comparison to chronic antigen exposure. Effector T cells are capable of secreting cytokines and have cytolytic activity. A portion of effector T cells can differentiate into memory T cells, capable of self-renewal, or undergo cell death. In contrast, exhausted T cell populations lack effector activity and undergo cell death. Progenitor T_ex_ cells express TCF1 (as well as other markers) and have enhanced proliferation potential. Terminal T_ex_ cells do not express TCF1 (and increased inhibitory receptor expression) and have reduced proliferation potential. Created with BioRender.com.

CTLs are very effective at removing foreign invaders in the body, such as during a viral infection. To do so, CTLs differentiate into their antigen-specific subtypes. Upon antigen recognition, naïve CD8^+^ T cells undergo clonal expansion and differentiate into effector CD8^+^ T cells, as shown in Figure 1. These immune cells are capable of cytokine secretion, have cytolytic activity, and function to target and clear virally infected cells. Once an infection is cleared from the body, the majority of these effector cells die leaving a small percentage (about 5%) of them to differentiate into long-lasting memory T cells (Tonnerre et al., 2021). This process in comparison with chronic antigen exposure is illustrated in Figure 1.

In the setting of a chronic antigen exposure, such as in cancer or chronic viral infections, the effector activity of CD8^+^ T cells is hindered. Even in tumors with a high mutational burden and the existence of a large amount of neoantigens, the immune system fails to completely clear all cancerous cells (Caushi et al., 2021; Belk et al., 2022). Thus, CD8^+^ T cells in the TME are thought of as being dysfunctional throughout tumorigenesis. This is mainly due to persistent antigenic stimulation during chronic infections or in the TME where CD8^+^ T cells lose their effector functions and are skewed towards an exhausted phenotype. There is now a wide belief that terminally exhausted CD8^+^ T cells are attributed to immune checkpoint blockade failures in the clinic, a point which will be discussed later in this review.

## An overview of CD8^+^ T cell exhaustion

T cell differentiation into an exhausted phenotype is a dynamic process, moving through a series of cellular states. Upon consistent antigenic stimulation either during chronic viral infections or cancer, effector CD8^+^ T cells begin to lose their proliferative capacity, cytokine production, and effector potential. They upregulate various inhibitory receptors (such as PD-1, CTLA4, Tim3, LAG3, *etc.*), alter known transcription factors (such as TCF1, T-bet, NFAT, TOX, Blimp-1, *etc.*), and incur a unique epigenetic state, all of which hinder their effector and cytolytic abilities (Tang et al., 2021). The upregulation of inhibitory receptors on exhausted CD8^+^ T cells, especially on progenitor exhausted CD8^+^ T cells (Tang et al., 2021), are targeted with ICBs and as a means of stimulating CTL effector functions of exhausted T cells. It is important to note that although chronic viral infection and cancer can both cause similar T cell differentiation into exhausted phenotypes as mentioned here, exhaustion in the TME is a subtly but importantly unique form of exhaustion which will be focused on throughout this review.

The different stages of exhausted CD8^+^ T cells differ greatly based on the expression levels of specific transcription factors and T cell inhibitory receptors. The progenitor exhausted T cells (T_ex_^prog1^) have a high proliferative potential with stem-like properties but low cytolytic ability (Jiang et al., 2021). In contrast, terminally exhausted CD8^+^ T cells (T_ex_^term^) have a low proliferative potential but high cytotoxicity (Jiang et al., 2021). The terminally exhausted CD8^+^ T cell subset is characterized by higher PD-1, Tim-3 and CD38 expression, and lower CXCR5, CD44, and Ly108 expression than progenitor CD8^+^ T_ex_ cells (Jiang et al., 2021). Furthermore, terminally exhausted CD8^+^ T cells overexpress the transcription factor TOX and do not express the transcription factors TCF1 and T-bet (Beltra et al., 2020). The differentiation into terminally exhausted CD8+T cells varies as well. For example, progenitor exhausted CD8^+^ T cells do not always turn into terminally exhausted subtype. Some reports suggest the existence of multiple intermediate cell subsets during this progression, if the progression occurs at all (Yang and Wang, 2021; Chen et al., 2022). To further outline the transcriptional heterogeneity of exhausted T cells, a new exhausted CD8^+^ T cell subset was identified recently which expresses unique natural killer (NK) cell related genes (i.e. *Klr*, *Fcg2rb*) and surface proteins (i.e. NK1.1, Ly49I, NKG2D) (Giles et al., 2022b).

The distinction between exhausted T cell subtypes is critical because although progenitor exhausted CD8^+^ T cells are responsive to ICBs, terminally exhausted cells are not (Kallies et al., 2020; Jiang et al., 2021). This distinction in part explains why such a large proportion of people fail to reach a complete response to ICB therapy, and why many people eventually progress with such treatments (Kallies et al., 2020; Jiang et al., 2021). One specific reason for this is that when antigen concentration remains high, exhausted CD8^+^ T cells which initially responded to checkpoint inhibitor therapy can become “re-exhausted” (Pauken et al., 2016). Pauken et al. demonstrated this finding in a mouse model of lymphocytic choriomeningitis virus (LCMV) with anti-PD-1 therapy. They found that CD8^+^ T cells failed to acquire memory after immune checkpoint therapy, even if exhausted T cells were reinvigorated (Pauken et al., 2016). This represents an important truism in cancer immunotherapy: once cells reach terminal exhaustion, it cannot be undone and likelihood of ICB resistance increases (Pauken et al., 2016).

Some immune receptors on T cells have been shown to modulate the expression levels of the inhibitory receptors that ICB therapy is trying to target, unveiling potential new approaches to immunotherapy. For example, our group demonstrated that the self-ligand immune receptor SLAMF7 was co-expressed with multiple inhibitory receptors (i.e. PD-1, Tim-3, LAG3) and when SLAMF7 signaling was induced in CD8^+^ T cells, the expression of PD-1, Tim-3, and LAG3 inhibitory receptors were upregulated (O’Connell et al., 2021). Also, SLAMF7 activation resulted in enhanced expression of several T cell exhaustion-promoting transcription factors and epigenetic regulators (O’Connell et al., 2021). We further demonstrated that presence of SLAMF7 on tumor-associated macrophages (TAMs) was necessary for TAMs to induce CD8^+^ T cell exhaustion during their cross-talk with CD8^+^ T cells (O’Connell et al., 2021). Accordingly, SLAMF7 expression on certain TAMs may be a prognostic factor in clear cell renal cell carcinoma (ccRCC) (O’Connell et al., 2021).

## Key transcription factors and chromatin modifiers regulating T cell exhaustion

It is now widely recognized that exhausted CD8^+^ T cells represent a distinct state in CD8^+^ T cell differentiation and, as such, have a unique profile of transcription factors (TFs) and epigenetic factors that drive and enforce this state *via* epigenetic remodeling and other mechanisms (Sen et al., 2016; Yao et al., 2019; Ranzoni et al., 2021). Understanding the mechanisms that regulate the expression of specific TFs, their temporal dynamics during the process of T cell exhaustion, and the cellular processes regulated by each TF has critical implications. These details help us not only understand the complex process of exhaustion, but also unveil how to pharmacologically alter this process for clinical benefit. A myriad of TFs have been implicated in regulating T cell exhaustion, some better defined than others, and a complete in-depth review of each can be found elsewhere (Wherry and Kurachi, 2015). There are also a number of TFs suspected to regulate exhaustion which have not yet been formally investigated, including E2F2 (Giles et al., 2022a), and likely others. Our group recently found that the immune cell surface receptor SLAMF7 is capable of regulating some of the critical exhaustion-defining TFs and epigenetic modifiers that regulate different aspects of T cell exhaustion (O’Connell et al., 2021). Below we detail what is currently known about each of these TFs and chromatin modifiers and how they regulate CD8^+^ T cell exhaustion programs.

### TOX

TOX has been described as the “master regulator” of T cell exhaustion (Alfei et al., 2019; Khan et al., 2019; Scott et al., 2019; Yao et al., 2019). However, the precise role TOX plays in T cell exhaustion is more nuanced and involves interplay with other TFs and temporal expression dynamics throughout T cell differentiation. As a brief review, CD8^+^ T cell exhaustion is not a binary state cells enter, but rather a continuous developmental trajectory which was definitively separated into four cell states by Beltra et al. (2020). Two of these cell states, T_ex_^prog1^ and T_ex_^prog2^, are progenitor states with stem-like capabilities. On the opposing end of the exhaustion developmental spectrum are the T_ex_^int^ and T_ex_^term^ states, which represent progressively more dysfunctional CD8^+^ T cells with T_ex_^term^ having a terminal and irreversible phenotype as reviewed here and in the literature (Beltra et al., 2020).

During the progression of CD8^+^ T cells towards terminal exhaustion, expression of TOX gradually increases along with a concomitant decrease in the transcription factor TCF1 (Scott et al., 2019; Beltra et al., 2020). This transition from TCF1 expression to TOX expression is critical in defining these divergent CD8^+^ T cell states. TCF1 is a well-established regulator of stem cell capabilities in CD8^+^ T cells (Siddiqui et al., 2019) and TOX functions to enforce the terminal exhaustion phenotype, while repressing the terminal effector state (Khan et al., 2019). Specifically, TOX epigenetically reinforces exhaustion programs *via* chromatin remodeling at promoters and enhancers of various genes driving T cell exhaustion. One way it does this is by inducing histone H3 and H4 acetylation and DNA methylation (Zeng et al., 2020).

There are a number of signals which induce TOX expression and this transition from stem-like CD8^+^ T cells to exhausted cells, including: repetitive TCR stimulation (Blank et al., 2019; Philip and Schietinger, 2019), IL-12 (Maurice et al., 2021), calcineurin (Khan et al., 2019), NFAT2 (Khan et al., 2019), STAT3 (Yao et al., 2019), SLAMF7 signaling (O’Connell et al., 2021), and likely others yet to be discovered. A number of these signals (calcineurin, NFAT2, and STAT3) are secondary to primary stimuli such as TCR activation (calcineurin and NFAT2) and SLAMF7 activation (STAT3 (O’Connell et al., 2021)). TOX expression in T cells does not itself induce exhaustion, nor is it a marker of T cell exhaustion. Indeed, increased TOX expression has been noted in PD-1+CD39^+^CD8^+^ T cells and EMRA CD8^+^ T cells from healthy control subjects (Balança et al., 2021; Maurice et al., 2021; Tallón de Lara et al., 2021). Furthermore, we and others have noted that TOX is transiently upregulated in T cells upon TCR stimulation (Khan et al., 2019; Maurice et al., 2021). Together, these findings suggest that TOX can be transiently expressed in CD8+T cells without committing them towards exhaustion, in a manner analogous to FOXP3 which is also transiently upregulated during CD4^+^ T cell TCR stimulation (Wang et al., 2007). Additionally, the expression levels of TOX vary significantly between terminally exhausted CD8^+^ T cells and any other T cell subset expressing TOX (Alfei et al., 2019; Khan et al., 2019; Scott et al., 2019; Yao et al., 2019). TOX expression also increases in CD8^+^ T cells along the exhaustion developmental trajectory defined by Beltra et al. (Beltra et al., 2020), with T_ex_^term^ having a TOX^high^ phenotype. This data further confirms that high TOX expression is indicative of true CD8^+^ T cell exhaustion, as can be appreciated in the difference in TOX expression noted between tumor infiltrating lymphocytes (TILs) and *in vitro* stimulated T cells (Khan et al., 2019; Giles et al., 2022a).

### T-bet

The T-box family member transcription factor T-box-expressed-in-T-cells (T-bet) has been implicated in playing an important role in the regulation of CD8^+^ T cell exhaustion, and it’s mechanism has recently been described. T-bet has well established roles in Th1 CD4^+^ T cell polarization (Szabo et al., 2000) and promoting T cell effector functions (Sullivan et al., 2003). It accomplishes such roles through binding T-box DNA elements leading to control of gene expression programs promoting these activities. More recently, roles for T-bet in the regulation of other cellular phenotypes in non-T immune cells have been described, such as its role in isotype switching and propagation of an antigen-experienced subset of B cells (Knox et al., 2019). Work by McLane et al. identified that T-bet antagonizes terminal exhaustion in CD8^+^ T cells *via* competition with another TF linked to T cell exhaustion, called Eomes (McLane et al., 2021). While both TF’s can bind to T-box sites in the promoter region of *Pdcd1* and block its expression, Eomes does so very weakly while T-bet strongly prevents PD-1 expression (McLane et al., 2021). McLane et al. identified that T-bet and Eomes compete for the same T-box binding domain in the promoter of *Pdcd1* and other exhaustion-linked genes, resulting in competition between the two. Therefore, the fraction of nuclear-localized T-bet *versus* Eomes plays a strong role in regulating CD8^+^ T cell exhaustion (McLane et al., 2021). Another group has made similar findings in humans with chronic HIV infection (Buggert et al., 2014). Work from Beltra et al. further supports the hypothesis that high nuclear T-bet expression antagonizes terminal exhaustion. They discovered that Eomes expression is highest in terminally exhausted (T_ex_^term^) cells and that these same cells have lower T-bet (Beltra et al., 2020). Accordingly, the T-bet^high^ PD-1^mid^ subset of CD8^+^ TILs is the subset capable of reinvigoration by checkpoint blockage, while the Eomes^high^ subset is not (Wherry and Kurachi, 2015). These studies on the role of T-bet in CTL exhaustion highlight the need to measure sub-cellular localization of many of these various proteins and TF’s linked to exhaustion. Thus, identifying appropriate roles for each in the context of exhaustion cell biology and revealing that merely measuring expression levels is likely not enough.

### Blimp-1

Blimp-1 (which is encoded by *prdm1)* was one of the first TF’s to be linked to T cell exhaustion (Wherry et al., 2007; Shin et al., 2009) and is thus one of the best characterized. Blimp-1 was first identified as a TF critical in the development of plasma cells (Shaffer et al., 2002) and has since been identified as a regulator of various functions in additional immune cell subsets (Nadeau and Martins, 2022). Blimp-1 primarily functions as a transcriptional repressor by either competing with other TFs for binding to specific DNA regulatory regions or by recruitment of other chromatin-modifying factors to specific genes (Nadeau and Martins, 2022). However, there are occasional instances where Blimp-1 can function as a transcriptional activator, as in the case of its regulation of *SLAMF7* (Kim et al., 2016).

The association of Blimp-1 with T cell exhaustion occurred after it was discovered that it was upregulated in T cells expressing multiple inhibitory receptors (Shin et al., 2009) and, more recently, in terminally exhausted CD8^+^ T cells (Yao et al., 2019). Conditional deletion of *prdm1* in activated CD8^+^ T cells *via* a granzymeB-Cre driver, so as to remove the possibility of *prdm1* deletion interfering with T cell development, revealed lower levels of multiple inhibitory receptors in *prdm1* cKO CD8^+^ T cells (Shin et al., 2009). It is important to note that while the *prdm1* cKO CD8^+^ T cells displayed decreased levels of multiple inhibitory receptors such as PD-1, 2B4, LAG3, TIGIT, and CD160, changes in PD-1 and LAG3 were minimal and 2B4 expression changes were the most pronounced (Shin et al., 2009; Zhu et al., 2017). 2B4 (*SLAMF4*) is a member of the SLAM family of receptors. Considering Blimp-1 has been shown to regulate the expression of another SLAM family member (*SLAMF7*) (Kim et al., 2016), and SLAMF7 has been shown to regulate Blimp-1 (O’Connell et al., 2021), this suggests Blimp-1 may preferentially regulate T cell exhaustion *via* SLAM signaling networks over that of other T cell inhibitory receptors.

It was also found that mice with conditional deletion of *prdm1* in CD8^+^ T cells controlled LCMV infection more effectively than WT mice (Shin et al., 2009), and that anti-CD19 CAR-T cells lacking Blimp-1 have improved *in vivo* tumor control (Yoshikawa et al., 2022). This data confirms the role for Blimp-1 in CD8^+^ T cell responses *in vivo*. These findings further support the theory that Blimp-1 regulates T cell exhaustion and effector functions, in conjunction with findings that high Blimp-1 expression in T cells inhibits IL-2 production (Gong and Malek, 2007).

Mechanistically, Blimp-1 drives T cell exhaustion in much the same way that it regulates cell states in other cell types, *via* regulation of chromatin accessibility to key genes important in driving functional T cell dysfunction (Yoshikawa et al., 2022). As a testament to how critical high Blimp-1 expression is in enforcing T cell exhaustion, Wu et al. demonstrated that Blimp-1 expression must be repressed by TCF1, the master TF regulating CD8^+^ T cell stemness (Wu et al., 2016). As discussed above, TCF1 functions in an inverse manner to TOX (the master positive regulator of exhaustion). Thus, the finding that TCF1 must directly antagonize both TOX and Blimp-1 demonstrates the importance of Blimp-1-controlled transcriptional programs in CD8^+^ T cell exhaustion.

### EZH2

The histone methyltransferase enhancer of zeste 2 polycomb repressive complex 2 subunit (EZH2) is a critical member of the polycomb repressive complex and has well-studied roles across developmental biology. Its importance in organismal development is exemplified by the fact that homozygous deletion of *EZH2* is embryonic lethal (O’Carroll et al., 2001), and necessitates conditional knockout models for its *in vivo* study. More recently, the contribution of EZH2 to immune cell biology has been investigated with a number of studies showing complex and important roles for this chromatin modifier in T cell biology (He et al., 2017; Goswami et al., 2018; Wang et al., 2018; Li et al., 2020; Stairiker et al., 2020). EZH2 functions as a methyltransferase which is capable of mediating di- and tri-methylation of histone H3 at lysine 27 (H3K27) on the promoter of target genes resulting in heterochromatin formation and repression of gene expression (Stairiker et al., 2020). In addition to its methyltransferase activity, EZH2 has been detected in the cytosol and shown to regulate actin polymerization and cell signaling (such as TCR signaling) (Su et al., 2005)].

Currently, EZH2 has been linked to memory T cell responses (He et al., 2017; Li et al., 2020), regulatory T cell responses in the TME (Goswami et al., 2018), altered T cell inhibitory receptor expression (He et al., 2017), and T cell differentiation towards an exhaustion phenotype (O’Connell et al., 2021). EZH2 has pleotropic effects in T cells as evidenced by the numerous above-mentioned roles of this TF and the variety of pathways it is known to cooperate in. For example, one mechanism by which EZH2 may be able to induce T cell exhaustion is *via* STAT3 phosphorylation, as demonstrated in part by our group and others (Kim et al., 2013; O’Connell et al., 2021). STAT3 has previously been shown to induce PD-1 expression, limit T cell effector functions, and promote formation of regulatory T cells, which together can drive T cell exhaustion (O’Connell et al., 2021). On the contrary, active EZH2 has also been shown to alter chromatin at the *prdm1* locus resulting in decreased Blimp-1 levels which may temper T cell exhaustion (He et al., 2017). To further complicate matters, EZH2 is able to regulate CD8^+^ T cells *via* activation of *Id3* and inhibition of *Id2* and *Eomes* (a TF which also plays an important role in T cell exhaustion and has been reviewed elsewhere (Li et al., 2018)) which together promote memory T cell formation and tumor control (He et al., 2017).

The key to EZH2’s diverse T cell regulatory abilities is its phosphorylation status, which determines if this chromatin modifier is active or not. When EZH2 is phosphorylated (*via* AKT), it is inhibited and unable to bind to and remodel chromatin and induce memory T cell formation (He et al., 2017). Additionally, signaling through the SLAMF7 cell surface receptor is capable of robustly inducing EZH2 levels in CD8^+^ T cells (O’Connell et al., 2021), but if this also alters EZH2 phosphorylation remains to be seen. Countering the induction of EZH2 expression in T cells by SLAMF7 signaling (which can be prevalent in the TME of certain cancers (O’Connell et al., 2021)) are unknown factors in the TME which negatively regulate EZH2 levels (Stairiker et al., 2020). Dissecting the dominant role of EZH2 in T cells in the TME is complicated by the above-mentioned pleotropic effects of this chromatin modifier, and also the fact that most studies on EZH2 have been performed in mice. As efforts move forward to modulate EZH2 for anti-tumor benefit with small molecule inhibitors (Kim and Roberts, 2016; Adema and Colla, 2022), there will need to be human-centric studies on the role of EZH2 in human TILs. The need to perform intranuclear staining for EZH2 has likely been a major factor contributing to the lack of human TIL EZH2 research, but as our group has shown (O’Connell et al., 2021), it is possible to optimize staining for this chromatin modifier.

### YY1

YY1 (Yin Yang 1) is a ubiquitously expressed TF harboring a large array of diverse and often opposing regulatory abilities (Gordon et al., 2006). YY1’s ability to regulate various cellular processes comes from its DNA binding function, where it serves as a TF capable of direct or indirect regulation of gene expression. Most early studies on YY1 focused on its function in cancer cells, however, more recent studies have highlighted additional roles as a TF capable of intrinsically modulating immune cell function (Gordon et al., 2006; Balkhi et al., 2018).

With regard to T cell exhaustion, YY1 has been found to control multiple processes during this phenomenon including modulation of inhibitory receptors and cytokine production (Balkhi et al., 2018). Specifically, it was found to drive transcription of PD-1, LAG3, and Tim3 in chronically stimulated CD8^+^ T cells *in vitro* (Balkhi et al., 2018). Additionally, YY1 was noted to induce T cell dysfunction *via* repression of T cell-intrinsic IL-2 production in a mechanism involving recruitment of EZH2 to the IL-2 promoter (Balkhi et al., 2018). As lack of IL-2 during active T cell proliferation is known to cause T cell dysfunction (Ross and Cantrell, 2018), this was thought to be a major mechanism whereby YY1 controls T cell exhaustion. Similar to Blimp-1, YY1 is also known to regulate SLAMF7 expression, although YYA does this *via* direct transcriptional repression (Dongre et al., 2013). Likewise, SLAMF7 can induce YY1 expression (O’Connell et al., 2021). This suggests the presence of a negative feedback loop involving YY1 and SLAMF7, although each likely only contributes to a fraction of the many regulatory signals each protein receives during normal immune cell function (O’Connell et al., 2021). Since YY1 has a well described role in regulating NFκB function (Gordon et al., 2006; Balkhi et al., 2018), its immune cell-modifying functions can be partially attributed to this mechanism as well. However, like many of YY1’s functions, its regulation of NFκB is complex (Gordon et al., 2006; Balkhi et al., 2018) and dissecting these mechanisms may be difficult.

## Epigenetic regulation of T cell exhaustion

Recent studies have demonstrated that T cell exhaustion is commonly associated with significant and unique genome-wide epigenetic remodeling programs which differentiate T_ex_ cells from naïve, effector, and memory T cells (Pauken et al., 2016; Sen et al., 2016; Philip et al., 2017). These epigenetic programs are associated with changes in expression of specific TFs, histone modifications, DNA methylation, and chromatin accessibility. For example, multiple epigenetic profiling studies in tumor infiltrating T_ex_ cells demonstrated altered chromatin accessibility in thousands of differentially accessible regions, including decreases in chromatin accessibility of genes associated with effector T cell differentiation, such as IFNγ, TNFα, IL-2, KLRG1, and others, and increases in chromatin accessibility of genes associated with T cell exhaustion, such as PD-1, Tim3, LAG3, TIGIT, TOX, Tcf7, NFAT, and others (Sen et al., 2016; Abdel-Hakeem et al., 2021; Giles et al., 2022a; Belk et al., 2022). These changes in chromatin accessibility were also associated with extensive transcriptional alteration in T_ex_ cells. Importantly, the transcriptional programs and chromatin profiles of T_ex_ cells were largely conserved between murine T_ex_ cells of LCMV-infected mice and human T_ex_ cells derived from cancer and chronically-infected patients (Bengsch et al., 2018; Pritykin et al., 2021), revealing common differentiation programs and regulatory mechanisms. These results also demonstrate that T cell alteration in tumors and chronic infections follows a shared epigenetic differentiation trajectory. In addition to having a similar transcriptional and chromatin profile, studies using murine tumor models identified CD38 and CD101 as differentiating markers for T_ex_ reprogrammability in PD-1 high CD8^+^ T cells. Specifically, PD-1^Hi^CD101^Low^CD38^Low^ was a plastic therapeutically reprogrammable chromatin state and PD-1^High^CD101^High^CD38^High^ was a fixed dysfunctional state of exhausted CD8+T cells (Philip et al., 2017). These findings demonstrate that the epigenetic state of T_ex_ cells is highly regulated and heterogenous, and this state governs the phenotype and function of T_ex_ cells, as well as their response to ICB therapy (Gyorki et al., 2009).

It is now well appreciated that the terminal epigenetic state of T_ex_^term^ is fixed and irreversible, even after reactivation, curative therapy, or following ICB therapies (Pauken et al., 2016; Hensel et al., 2021; Tonnerre et al., 2021; Yates et al., 2021). For example, unlike memory CD8^+^ T cells which robustly expanded upon restimulation, CD8^+^ T_ex_^term^ cells from anti-PD-1-treated tumor-bearing mice failed to respond to secondary stimuli (Pauken et al., 2016). These results were associated with persistent PD-1 upregulation and a specific DNA methylation signature at the *Pdcd1* and other gene loci (Pauken et al., 2016). Also, anti-PD-1 therapy was able to only change 10% of the epigenetic landscape to T_ex_^term^ cells, implicating that the fixed chromatin state of T_ex_^term^ cells is a major limitation for anti-PD-1 therapy (Pauken et al., 2016). Similarly, the chromatin accessibility of T_ex_^term^ cells derived from HIV- and HCV-infected patients remained unchanged before and after resolution of the infection, and in particular at the *Tox* and *Pdcd1* loci, confirming a fixed epigenetic state in T_ex_ cells after they reach terminal exhaustion (Abdel-Hakeem et al., 2021; Yates et al., 2021). Additionally, and due to the nature of the fixed chromatin state of T_ex_^term^ cells, when T_ex_ cells from cured HCV patients were re-challenged with HCV virus, robust upregulation of T cell exhaustion-related genes like PD-1 occurred (Abdel-Hakeem et al., 2021). This phenotype was also associated with reduced expression of genes that regulate effector T cell activity, which further impaired their effector functions. These results necessitate the need to develop novel therapeutic approaches that increase the epigenetic plasticity of T_ex_ cells. It is important to note that the T_ex_ cell differentiation state, which is enforced at the epigenetic level, defines specific subsets of T_ex_ cells. For example, T_ex_^prog1^ (Ly108+CD69^+^) and T_ex_^int^ (Ly108+CD69^−^), which can proliferate and self-renew with ICB therapy (Gyorki et al., 2009) have accessible chromatin in various gene loci that is associated with T cell stemness, such as the Ly108 (*SLAMF6*) and TCF1 (*Tcf7)* gene loci (Zehn et al., 2022). In contrast, the terminally exhausted ((Ly108^−^CD69^+^); T_ex_^term^) T cell subset, which does not respond to ICB therapy, has accessible chromatin at the gene loci of NR4A, EOMES, and TOX TFs and PD-1, TIM-3, LAG3, CD101, and CD38 T cell inhibitory receptors (Hudson et al., 2019). These data reveal intricacies within the exhausted T cell population, and their implications in cancer progression and tumor evasion of ICB therapy.

## Technologies for studying epigenetics

### ATAC-seq

**FIGURE 2:**
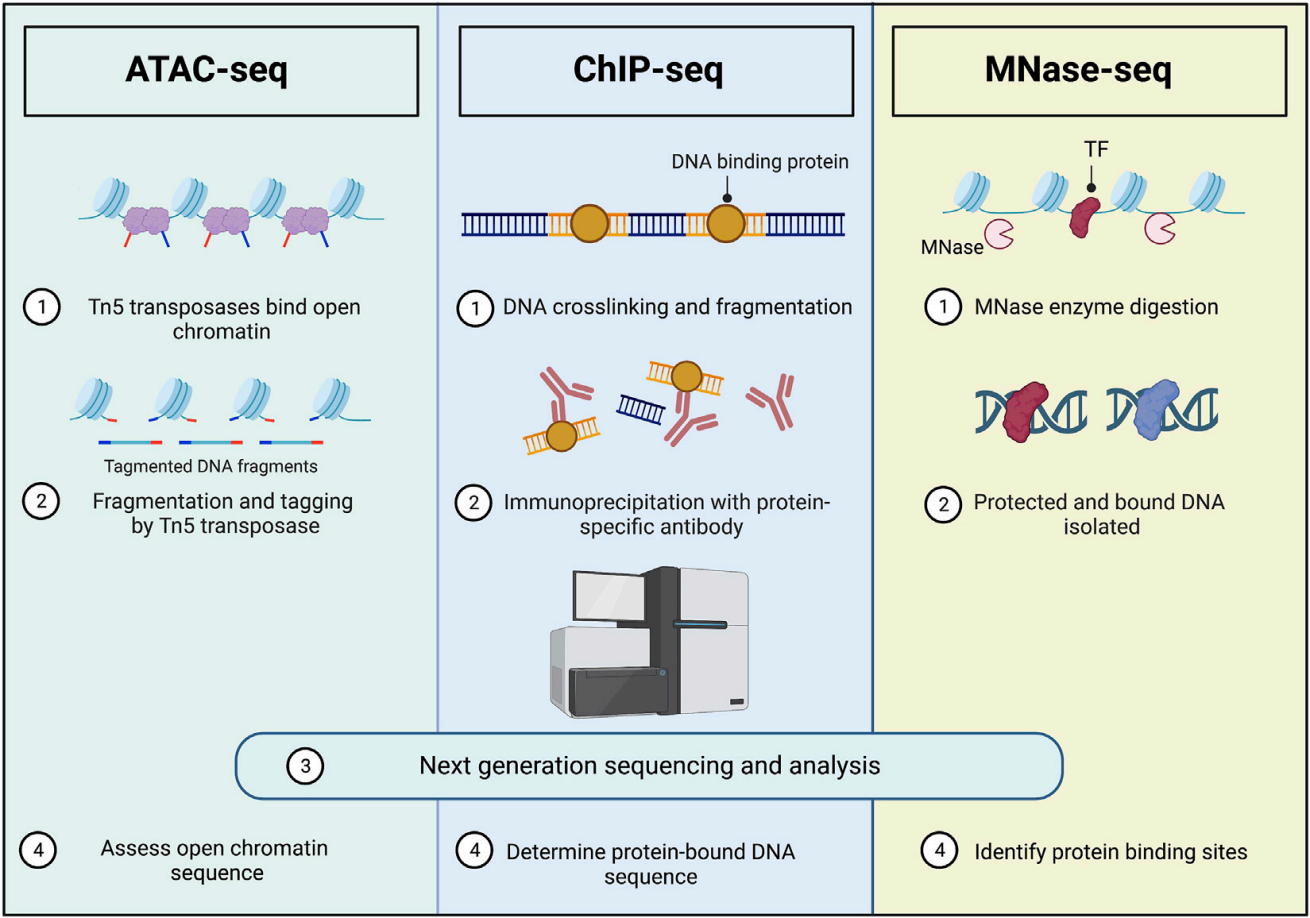
Common methods for studying epigenetic modifications of chromatin. ATAC-seq, ChIP-seq, and MNase-seq have different mechanisms for DNA isolation, but all result in sequencing of specific DNA structures to identify epigenetic modulations. ATAC-seq involves the use of Tn5 to assess the location and sequence of open chromatin, ChIP-seq assesses the sequence of protein-bound DNA through crosslinking and fragmentation, and MNase identifies protein binding sites on DNA using an MNase enzyme digestion which leaves bound DNA to be isolated and sequenced. These techniques can be completed on bulk cells or single cells as described in this review. Created with Biorender.com and adapted from Chen et al., 2022.

Various unique techniques have been developed and optimized to study the epigenetic profile of exhausted T cells, with one of the most prominent being the assay for transposase-accessible chromatin with sequencing, or ATAC-seq. Figure 2 illustrates ATAC-seq and the other methods that are discussed in this review. This specific technique involves the cleavage of chromatin by Tn5, which directly cuts only accessible chromatin (Belk et al., 2022). ATAC-seq therefore profiles only DNA fragments of genomic locations which are accessible for transcription. This technology allows one to identify the genomic accessibility at a precise location. Indeed, ATAC-seq transposes native chromatin for epigenomic profiling in a fast and sensitive way, and is thus a powerful technique for profiling the epigenetic regulation of T cell exhaustion. Recently, advances have made it possible to conduct ATAC-seq on single cells (Fang et al., 2021; Ranzoni et al., 2021)and in a spatial context (Thornton et al., 2021), making the unique cell populations and architecture of TME possible to profile.

Specific to T cell exhaustion, studies found that exhausted T cells had similar chromatin accessibility in different tumor models (Pritykin et al., 2021). This work was done by analyzing over 300 assays of ATAC-seq and RNA-seq. Authors found, through ATAC-seq, that functional and dysfunctional T cells diverged from a progenitor-like population of T cells (Pritykin et al., 2021). Multiple other studies have been done using the ATAC-seq technique, revealing different and specific chromatin accessibility changes in effector T cells, exhausted T cells, and memory T cells (Sen et al., 2016). Studies also have shown significant differences in regulatory region patterns between these different T cell populations with ATAC-seq, overall supporting its clear importance. Thorough reviews have been done on the application of ATAC-seq in the tumor microenvironment and we recommend their review for more information (Sen et al., 2016; Chen et al., 2022).

### ChIP-seq and MNase-seq

Other techniques can be used to locate specific histone modifications or DNA-binding protein locations. Chromatin immunoprecipitation with sequencing (ChIP-seq) (Figure 2) is a technology for identifying genome-wide DNA binding of a protein of interest (or chromatin modification) and involves crosslinking DNA with all bound proteins, fragmenting the DNA, immunoprecipitation, followed with sequencing of the pulled-down DNA (Mikkelsen et al., 2007; Belk et al., 2022). Antibody-tethered micrococcal nuclease (MNase-seq) (Figure 2) is an advancement of ChIP-seq, also known as the CUT&RUN method, where the chromatin is cut directly next to the bound protein, allowing the DNA fragments to move out of the nucleus where they can then be isolated and sequenced (Skene and Henikoff, 2017; Belk et al., 2022). The main advantage of this technique is that is does not require fixation and can be done with fewer cells than ChIP-seq, allowing for sequencing of rare cells. Similarly to ATAC-seq, ChIP-seq can now be done on single cells, advancing its ability to detect changes in small immune populations (Grosselin et al., 2019). Furthermore, HI-ChIP is a new “protein-centric” chromatin conformation technique which improves readability and requires a lower sample size than ChIP-seq (Mumbach et al., 2016).

### Multiomic approach

The above mentioned epigenetic techniques are able to be performed with other transcription- and proteome-wide profiling techniques as well, constituting a multiomic method. This allows for profiling multiple layers of cell processes at the same time in a single cell. A couple examples include nucleosome occupancy methylome sequencing (NOME-seq), which measures DNA methylation and chromatin accessibility at the same time, showing the nucleosome position along with DNA methylation in the chromatin (Mehrmohamadi et al., 2021). However, these assays are limited to the CpG frequency in the genome. To account for this limitation, one can alternatively combine ATAC-seq with bisulfite conversion in a technique known as EpiMethylTag to study DNA methylation and chromatin accessibility on the same DNA molecule (Mehrmohamadi et al., 2021). Although most of these approaches are applicable to single-cell sequencing, they are not as optimal for single cells due to low throughput (Mehrmohamadi et al., 2021). Non-etheless, the technology is improving quickly and will likely be applicable to single cells in a robust manner soon.

## T cell reprogramming

**FIGURE 3:**
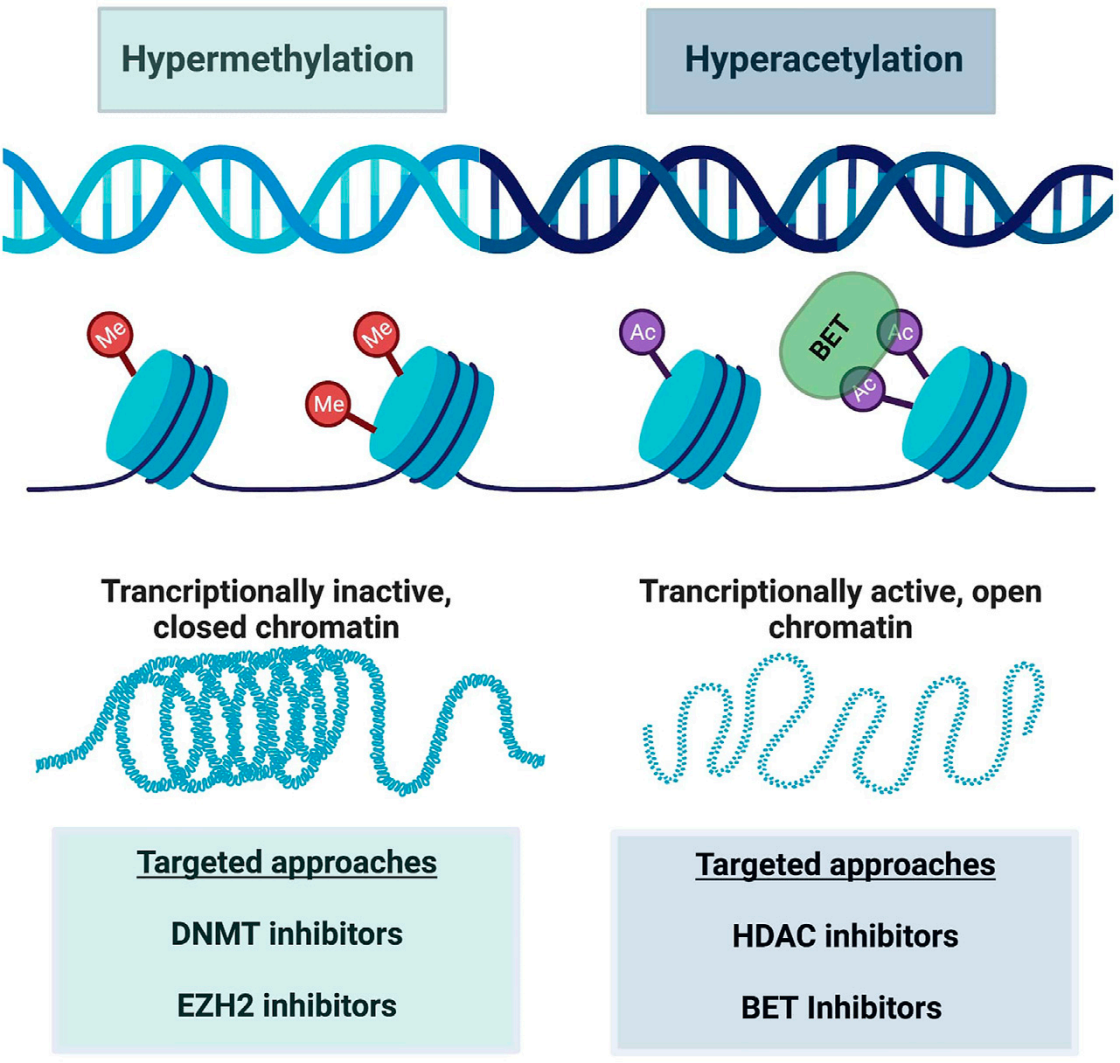
A schematic of major epigenetic alterations reinforcing CD8^+^ T cell exhaustion. Hypermethylation is characterized by increased methyl group attachment to DNA on chromatin, making the DNA transcriptionally inactive and closed. Hyperacetylation on the other hand acts in an opposite manner, where acetyl group binding causes chromatin to open and enhanced transcription of DNA. BET targeting to acetyl groups can also modify this transcription. These alterations are manipulated by various targeted drug approaches as listed in this figure. Created with BioRender.com.

As mentioned throughout this review, resistance to ICB and CAR-T cell therapy is a critical clinical problem requiring further research. Scientists have discovered that this resistance, at least in part, occurs due to epigenetic changes in T cell differentiation and in particular in exhausted CD8^+^ T cells. Thereby, it is hypothesized that certain epigenetic-targeting drugs could be applied to T_ex_ cells as a means of restoring stemness. Indeed, there are various classes of epigenetic modifiers, both in development and FDA approved, which will be discussed here along with their current utility. It is important to note that most of these epigenetic modifiers do so on a global scale, so specificity to a subset of immune cells in the tumor microenvironment is currently limited. Figure 3 demonstrates how these therapies can be conceptualized in addition to the altered epigenetic modifiers discussed here.

Broad-spectrum epigenetic inhibitors target genome-wide cancer specific gene expression and include inhibition of various targets. Specifically, inhibition of DNA methyltransferase (DNMT) (Chiappinelli et al., 2015; Giri and Aittokallio, 2019), histone deacetylase (HDAC) (Eckschlager et al., 2017; Lue et al., 2019), and bromodomain and extra-terminal motif proteins (BET) (Kong et al., 2021; Shorstova et al., 2021) are the most common. DNMT inhibitors target hypermethylated DNA regions, and thus transcriptionally inactive chromatin. This reversal of silenced gene transcription by loss of DNA methylation allows the genes to become active again, which is desirable in the setting of tumor suppressor gene transcriptional silencing. DNMT inhibitors have been used in treating blood malignancies for years (Gore, 2005). Specifically, decitabine and azacytidine, two DNMTi drugs, are approved for treating myelodysplastic syndrome (MDS) and acute myeloid leukemia (AML). The drugs’ activity towards malignant cells supports the importance of epigenetic regulation in tumors. More recently, DNMTi use has been found to affect T cell exhaustion (Wang et al., 2021). Wang *et al.* found that that decitabine epigenetically reprogrammed CAR-T cells, enhancing and prolongating their antitumor activity (Wang et al., 2021). Furthermore, other groups showed that DNMTi therapy reversed exhausted CD8^+^ T cells and enhanced their effector function in different cancer models (Chiappinelli et al., 2015; Roulois et al., 2015). DNMT inhibitor therapy with various different drugs are currently being tested in clinical trials for multiple hematologic and solid malignancies, most in combination with another agent such as chemotherapy, immunotherapy, or second epigenetic modifying drug (Gore, 2005; Hu et al., 2021; Baer et al., 2022).

HDAC inhibitors on the other hand, preserve acetyl groups on lysine residues in histone tails, allowing open chromatin configurations and thus favoring gene transcription (Laino et al., 2019). Indeed, HDAC inhibitors cause apoptosis and arrest tumor cell growth and potential angiogenesis. There are multiple HDAC isoforms, and some inhibitors target specific HDAC isoforms while others are global inhibitors (Laino et al., 2019). For example, selective HDAC6 inhibition with ACY-1215 and ACY-241 was efficacious towards melanoma patients’ T cells *ex-vivo*, harvested from either peripheral blood or tumor biopsies (Laino et al., 2019). Specifically, HDAC6 inhibitor increased T cell effector functions, reduced exhaustive markers, and in turn enhanced the killing of malignant cells (Laino et al., 2019). Importantly, higher frequencies of T effector memory cells remained after treatment with ACY-1215 as well (Laino et al., 2019). Chromatin accessibility was increased in genetic regions associated with such effector and memory T cell functions. Furthermore, McCaw et al. found that an HDAC inhibitor, specifically the HDAC1 inhibitor etinostat (ENT), promoted immune-modulatory molecule expression which in turn re-sensitized tumors to ICB (McCaw et al., 2019). These findings support that epigenetic targeting can reprogram exhausted T cells and enhance effector function of T cells once resistant to ICB.

Another global inhibitor of epigenetic processes are bromodomain and extra-terminal domain (BET) inhibitors. BET overexpression can contribute to carcinogenesis, such as in hyper-acetylation of proliferation-promoting genes or hyper-acetylation of oncogene enhances (Jin et al., 2021). BET inhibitors recognize and bind to acetylated lysine residues, altering transcriptional activation and chromatin remodeling (Shorstova et al., 2021). Indeed, mouse studies revealed that suppression of BET proteins reduced AML burden (Zhong et al., 2022). Moreso, the specific BET inhibitor JQ1 reversed T cell exhaustion from ICB therapy in AML T cells *in vitro* and *in vivo* (Zhong et al., 2022). This BET inhibitor also reduced PD-1 and Tim-3 expression and increased cytokine secretion by AML patient-derived T cells (Zhong et al., 2022). As exciting as this data is, whether BET inhibition can reverse exhaustion in CML *in vivo* models which are resistant to anti-PD-1 therapy remains to be seen, and would be an important next step. Using a chronic lymphocytic leukemia (CLL) model, Kong et al. did demonstrate that BET inhibition reversed CAR-T cell exhaustion as measured by multiple factors including reduced inhibitory receptor expression and increasing their proliferation abilities (Kong et al., 2021).

Other methods of epigenetic modulation include narrow spectrum inhibitors. One of these methods focuses on EZH2 inhibition, due to its methyltransferase activity. Wang *et al.* found that EZH2 depletion reduced MYC expression and hindered neuroblastoma and small cell lung carcinoma tumor growth (Wang et al., 2022). However, they found that this EZH2 inhibition was independent of its methyltransferase activity (Wang et al., 2022), revealing that EZH2 may play a role in cancer beyond its enzymatic epigenetic reprogramming abilities. In 2020, the first EZH2 inhibitor was FDA approved for patients with relapsed or refractory follicular lymphoma. To receive this treatment, patients require a confirmed EZH2 mutation, as the studies determined a higher response rate in patients with confirmed EZH2 mutation in comparison to WT (69% vs 35%) (Morschhauser et al., 2020). However, the therapy was found to be tolerable and produce reasonably efficacy independent of EZH2 mutation (Morschhauser et al., 2020). Thus, it could be argued that this could be an option for refractory lymphoma patients (or other B cell malignancies) without EZH2 mutation, given its reasonable efficacy and safety profile.

Different EZH2 inhibitors exist, most targeting the methyltransferase abilities of EZH2. For this reason, it has been proposed as a promising combination with cytotoxic chemotherapy, as it makes DNA more accessible for DNA-damaging agents (Adema and Colla, 2022). In theory, one could use a lower dose of cytotoxic drug with a largely not toxic EZH2 inhibitor. This would improve safety profiles of chemotherapy, especially in older individuals (Adema and Colla, 2022). Furthermore, it can be combined with other epigenetic modifiers for better results. For example, the combination of EZH2 inhibitor with HDAC inhibitor increased lymphoma cell line apoptosis (Lue et al., 2019). The impact of combination epigenetic reprogramming on CD8^+^ T cell exhaustion and possible synergistic effects *in vivo* are unclear and warrant further investigations.

Outside of targeting epigenetic enzymes, research has supported targeting important exhaustion-driving transcription factors, and preclinical results have been promising. For example, CAR-T cells with Nrf4A family proteins knocked out had improved tumor-directed efficacy in mouse models, and knockout of TOX improved CAR-T cell efficacy as well (Tang et al., 2021). Although there are no current TOX inhibitors, this would seem to be a viable option for reversing ICB resistance. Another option could be to use an upstream regulator of TOX. Specifically, a regulator such as SLAMF7 which regulates TOX expression as discussed above. Thus, we propose that epigenetic directed therapy in combination with a SLAMF7 inhibitor may be a way to reduce CD8+T cell terminal exhaustion and warrants future investigations.

## Discussion

It is no question that the discovery of checkpoint inhibitors and development of CAR-T cell-based therapy have drastically changed the way clinicians approach cancer treatment and management. However, despite great success, many tumors unfortunately remain insensitive to such therapies, with solid tumors remaining particularly challenging to treat *via* immune-based mechanisms. Recently, the discovery of epigenetic changes driving T cell differentiation toward exhaustion has unveiled that epigenetic factors reinforce CD8^+^ T cell exhaustion as well. These studies, together with known transcription factors driving CD8^+^ T cell exhaustion, support that the exhausted CD8^+^ T cell phenotype is at least in part due to epigenetic modulations. Thus, the utilization of epigenetic modifying drugs either as single agents or in combination with potent therapies might improve the effector and memory responses of CD8^+^ T cells.

As important as the understanding of epigenetic remodeling of T_ex_ cells is for the tumor immunology field, how these drugs will translate *in vivo* remains to be seen. For example, many of these drugs are global modifiers of specific epigenetic processes. Thus, they would need to be directed towards exhausted CD8^+^ T cells specifically to confirm these theories. Utilization of a drug-antibody conjugate for an inhibitory receptor highly expressed on exhausted CD8^+^ T cells, such as PD-1, may be one way to localize epigenetic modifying therapies to the T_ex_ immune cell subset in the TME. In practice, it may be more beneficial to epigenetically modify both cancer cells and T_ex_ cells, in which case a purely T cell-targeted approach would not be as desirable, but this remains to be seen. Not only may the inhibitors have effects on tumor cells, but they also can have off target effects on healthy cells and tissues unrelated to cancer. In addition, it is well-known that potent immune activation in the setting of cancer can trigger autoimmune-like reactions (Young et al., 2018). Thus, ensuring the therapy is selective for exhausted CD8^+^ T cells is an important limitation of current studies and should be further addressed.

This work leads one to wonder whether the combination of broad-spectrum epigenetic regulators in combination with ICB or CAR-T cells therapy would be optimal in cancer patients who do not respond to ICB alone, and some studies already support this hypothesis (Eckschlager et al., 2017; Li et al., 2018; McCaw et al., 2019; Wang et al., 2021; Zhong et al., 2022). Whether it is due to reversing CD8^+^ T cell exhaustion or some other factor remains to be seen. The lack of specificity with global epigenetic regulators may limit their efficacy and makes mechanistic understanding of the inhibitors in relation to exhaustion difficult to discern. This muddies identifying patients who will respond to the therapy *versus* those who will not. Currently, genetic testing of specific epigenetic regulators is being done on patients to predict their response to such inhibition. However, as we have learned from targeted therapy approaches, because a gene is altered does not mean that targeted inhibition will reverse the phenotype. Indeed, it is unclear whether patients without such mutations could also respond to therapy, which would support that these epigenetic therapies could be efficacious for a larger population. Given patients with reduced options, we propose that epigenetic approaches should be considered. The authors here believe that this is a field of study with great promise and may be a possible solution to ICB or CAR-T cell resistance. Further research is necessary to unweave the intricate epigenetic mechanisms in exhausted CD8^+^ T cells, improve specificity of epigenetic modifying therapy, and discover optimal approaches for reversing terminal CD8^+^ T cell exhaustion in the TME.
